# Epigallocatechin-3-Gallate-Loaded Liposomes Favor Anti-Inflammation of Microglia Cells and Promote Neuroprotection

**DOI:** 10.3390/ijms22063037

**Published:** 2021-03-16

**Authors:** Chun-Yuan Cheng, Lassina Barro, Shang-Ting Tsai, Tai-Wei Feng, Xiao-Yu Wu, Che-Wei Chao, Ruei-Siang Yu, Ting-Yu Chin, Ming Fa Hsieh

**Affiliations:** 1 Division of Neurosurgery, Department of Surgery, Changhua Christian Hospital, 135 Nanxiao St., Changhua City, Changhua County 500, Taiwan; 83998@cch.org.tw; 2 Department of Biomedical Engineering, Chung Yuan Christian University, No. 200, Zhongbei Rd., Zhongli Dist., Taoyuan City 320314, Taiwan; lassinabarro_dieron@yahoo.fr (L.B.); shant7707@gmail.com (S.-T.T.); taverenspirit85@gmail.com (T.-W.F.); ggg2004aaa@yahoo.com.tw (X.-Y.W.); shung804@yahoo.com.tw (R.-S.Y.); 3 Center for Minimally-Invasive Medical Devices and Technologies, Chung Yuan Christian University, No. 200, Zhongbei Rd., Zhongli Dist., Taoyuan City 320314, Taiwan; 4 Department of Bioscience Technology, Chung Yuan Christian University, No. 200, Zhongbei Rd., Zhongli Dist., Taoyuan City 320314, Taiwan; oe4213@gmail.com

**Keywords:** neuroprotection, neuroinflammation, Parkinson’s disease, catechin, L-α-phosphatidylcholine, phosphatidylserine

## Abstract

Microglia-mediated neuroinflammation is recognized to mainly contribute to the progression of neurodegenerative diseases. Epigallocatechin-3-gallate (EGCG), known as a natural antioxidant in green tea, can inhibit microglia-mediated inflammation and protect neurons but has disadvantages such as high instability and low bioavailability. We developed an EGCG liposomal formulation to improve its bioavailability and evaluated the neuroprotective activity in in vitro and in vivo neuroinflammation models. EGCG-loaded liposomes have been prepared from phosphatidylcholine (PC) or phosphatidylserine (PS) coated with or without vitamin E (VE) by hydration and membrane extrusion method. The anti-inflammatory effect has been evaluated against lipopolysaccharide (LPS)-induced BV-2 microglial cells activation and the inflammation in the substantia nigra of Sprague Dawley rats. In the cellular inflammation model, murine BV-2 microglial cells changed their morphology from normal spheroid to activated spindle shape after 24 h of induction of LPS. In the in vitro free radical 2,2-diphenyl-1-picrylhydrazyl (DPPH) assay, EGCG scavenged 80% of DPPH within 3 min. EGCG-loaded liposomes could be phagocytized by BV-2 cells after 1 h of cell culture from cell uptake experiments. EGCG-loaded liposomes improved the production of BV-2 microglia-derived nitric oxide and TNF-α following LPS. In the in vivo Parkinsonian syndrome rat model, simultaneous intra-nigral injection of EGCG-loaded liposomes attenuated LPS-induced pro-inflammatory cytokines and restored motor impairment. We demonstrated that EGCG-loaded liposomes exert a neuroprotective effect by modulating microglia activation. EGCG extracted from green tea and loaded liposomes could be a valuable candidate for disease-modifying therapy for Parkinson’s disease (PD).

## 1. Introduction

When the nervous system is damaged or infected, microglia cells are activated and transformed to be branched, resulting in excessive expression of a large amount of pro-inflammatory cytokines such as tumor necrosis factor-α (TNF-α), interleukin-1β (IL-1β), interleukin 6 (IL-6), and inflammatory mediators such as nitric oxide (NO) and reactive oxygen species (ROS). Finally, nerve cells are damaged, degenerated, or die from these inflammatory mediators. It is recently found that in the brain of patients with neurodegenerative diseases such as Parkinson′s disease (PD), Alzheimer′s disease, Huntington′s disease, and Creutzfeldt-Jakob disease, large amounts of microglia cells are activated and over-expressed [1,2,3]. Epidemiologically, PD’s cause is mostly linked to the neuroinflammatory reaction. The inflammatory mediators resulting, such as TNF-α, IL-1β, IL-6, NO, and ROS, are found in the striatum of the brain [1,4,5,6,7]. The degradation of dopaminergic neurons can be regulated by microglia cells [8].

The neuroinflammation process that causes PD is preceded by primary damage of neurons caused by environmental toxins, including rotenone [9], lipopolysaccharide (LPS) [5,7], and the effects of abnormal protein accumulation [10]. The damage will cause lesions and even apoptosis of dopaminergic neurons. Then microglia cells are activated to release cytokines, resulting in inflammation and death of the neurons and finally leading to PD.

When the microglia cells are stimulated by LPS, LPS binds to the surface receptor CD14 binding site of microglia cells. The LPS-CD14 complex is linked with MD2 linker through toll-like receptor-4 (TLR4) transmembrane proteins and then involved in multiple message transmission pathways generated by mitogen-activated protein kinases (MAPK) and activating transcription factors (nuclear factor-kappa B, NF-κB). After gene transcription [5,11,12], microglial cells release cytokines such as TNF-α and IL-1β, or express genes of inducible nitric oxide synthase (iNOS) and cyclo-oxygenase-2 (COX-2), resulting in the release of prostaglandins or NO. In addition, destructive ONOO free radicals are generated by combining superoxide anions produced from nicotinamide adenine dinucleotide phosphate (NADPH) oxidase with NO produced from iNOS, leading to the death of dopaminergic neurons [13]. Therefore, in this study, LPS was used to induce neuroinflammation of microglia cells as an in vitro model of PD.

Catechins are natural antioxidants that can prevent cell damage and provide many pharmacological benefits such as anti-tumor, anti-cancer, anti-aging, anti-pharmacological radiation, and free radical scavenging [14]. Green tea contains about 10% polyphenols by weight, including large amounts of a catechin called epigallocatechin gallate (EGCG). EGCG has the highest antioxidant activity and free radical scavenging capacity in all green tea catechins and can capture ROS to protect cells from the damage of oxidative stress [15]. EGCG also has high anti-inflammatory efficacy, which can effectively inhibit secretion of cytokines (TNF-α, IL-2, and IL-8) by macrophages [16], phosphorylation of Akt signaling proteins and IκB proteins in inflammatory pathways to reduce NF-κB expression, or AP-1 transcription by inhibiting phosphorylation of upstream MAPK proteins to balance COX-2 expression and reduce the production of pro-inflammatory cytokines [17].

Recently, EGCG was reported to be potentially therapeutic or prophylactic for PD due to suppressing active oligomers of α-synuclein (αS) [18]. EGCG also prevents αS aggregation in vitro [19,20,21], and cytoplasmic αS aggregation in dopaminergic neurons is one possible pathogenesis of PD leading to damage of dopaminergic neurons in substantia nigra [22]. Furthermore, EGCG can recover 1-methyl-4-phenyl-1,2,3,6-tetrahydropyridine (MPTP)-induced neurochemical or functional damage and regulate ferroportin in substantia nigra and reduce oxidative stress [23]. EGCG also has neuroprotective and immune-protective effects in MPTP-treated mice and can modulate neuroinflammation and protect dopaminergic neuron loss in MPTP-induced PD [24].

The anti-inflammation effects of EGCG were investigated. EGCG suppressed LPS-induced NO production and expression of iNOS in BV-2 microglial cells. EGCG can effectively inhibit the expressions of pro-inflammatory cytokines such as TNF-α and IL-1β in BV-2 cells [25]. EGCG pretreatment of human macrophages significantly inhibited LPS-induced expression of pro-inflammatory cytokines such as TNF-α, IL-1β, and IL-6 [26]. In addition, post-treatment of EGCG on LPS-injured mice decreased production of a pro-inflammatory cytokine through modulating the TLR4-NF-κB pathway [27]. Moreover, poly(lactide-co-glycolide) (PLGA) microspheres loaded with EGCG and optimized by the addition of β-cyclodextrin (β-CD) could effectively suppress NO production from BV-2 cells in the in vitro model of murine BV-2 microglial cells stimulated by LPS, indicating the microspheres can suppress inflammation of activated microglial cells [28].

Although green tea is a daily drink, the efficacy of catechins is ineffective due to low oral bioavailability; thus, effective pharmaceutical dosage forms are required. Nano drug carrier has the benefits of avoiding premature metabolism, extending drug action time, and targeting drug delivery. Therefore, this study intends to develop liposomes containing phosphatidylcholine (PC) and phosphatidylserine (PS), similar components to the cell membrane, as anti-inflammatory dosage forms. EGCG extracted from green tea leaves was loaded in liposomes to slow down inflammatory reaction in microglia cells induced by LPS. The therapeutic effect of EGCG-loaded liposomes on the in vivo model of PD for neuroprotection was also evaluated.

## 2. Results

### 2.1. Extraction of EGCG

#### 2.1.1. EGCG Extract

The epigallocatechin-3-gallate characterization data can be found in the Supplementary File (Figures S1 and S2).

#### 2.1.2. Various Formulations of EGCG

In Table 1, the average particle diameters of placebo PS-, PS-EGCE-, and PS-EGCG-VE-liposomes were smaller than that of placebo PC-, PC-EGCE-, and PC-EGCG-VE-liposomes, respectively. Because PC is neutral and PS is negatively charged [29], this indicates that the additive surface potential affected the particle size. Polydispersity index (PDI) of all liposomes was less than 0.22, indicating that the structure of liposomes in solution is stable. The negative charges of PS led to a repulsive force on the surface potential between PS-liposomes, avoiding aggregation and reducing the size of PS-liposomes.

The encapsulation efficiency/size of PS-containing liposomes described in Table 1 was larger/smaller than that of corresponding PC-containing liposomes [30]. The encapsulation efficiency of PC-EGCG-VE-liposomes and PS-EGCG-VE-liposomes was more extensive than that of PC-EGCG-liposomes and PS-EGCG-liposomes, respectively. This is because vitamin E is fat-soluble, embedded in a phospholipid bilayer membrane, and provides an antioxidant protectant for EGCG.

### 2.2. In Vitro Cell Analysis

#### 2.2.1. Cell Viability

**Figure 1:**
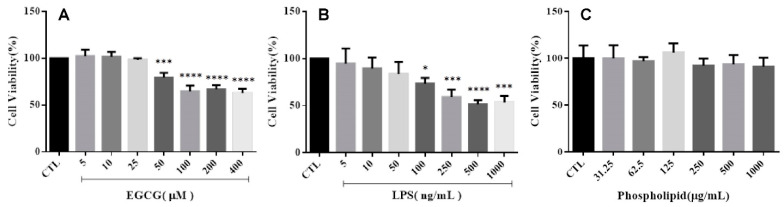
Cell viability of BV-2 cells treated by (**A**) EGCG, (**B**) lipopolysaccharide (LPS), and (**C**) placebo liposome (compared with control group (CTL), * *p* < 0.05; *** *p* < 0.001, **** *p* < 0.0001, *n* = 3).

The cell viability of cells treated with EGCG from 50 to 400 μM was significantly decreased compared to the control group (Figure 1A). In contrast, the viability of cells receiving 5 to 25 μM is similar to the control group cells’ viability. The concentration of EGCG used in this study was determined to be 25 μM. Similarly, the concentration of LPS used for induction of cell inflammation was determined to be 50 ng/mL according to Figure 1B. In Figure 1C, the cell viability of cells co-cultured with placebo liposomes of all concentrations was not statistically significant compared to the control group. Therefore, placebo liposomes are not cytotoxic to microglia cells.

#### 2.2.2. Cell Morphology

**Figure 2:**
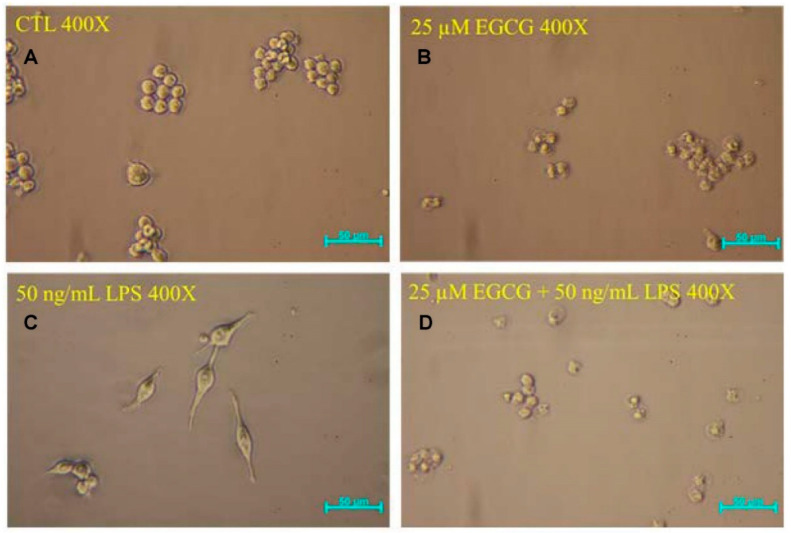
Morphology of BV-2 cells (**A**) of the control group, (**B**) treated with EGCG, (**C**) treated with LPS, and (**D**) treated with EGCG and then LPS.

Cell morphology of the control group (Figure 2A) and the morphology of cells treated with 25 µM EGCG (Figure 2B) were spherical, while the morphology of cells treated with 50 ng/mL LPS (Figure 2C) was spindle-shaped. However, the morphology of cells treated with 25 µM EGCG and activated by LPS (Figure 2D) was spherical. This denoted that EGCG can inhibit the activation induced by LPS. Therefore, the pretreatment of EGCG has an inhibitory effect on neuroinflammation, protecting the microglial cells from activation.

#### 2.2.3. NO Release

**Figure 3:**
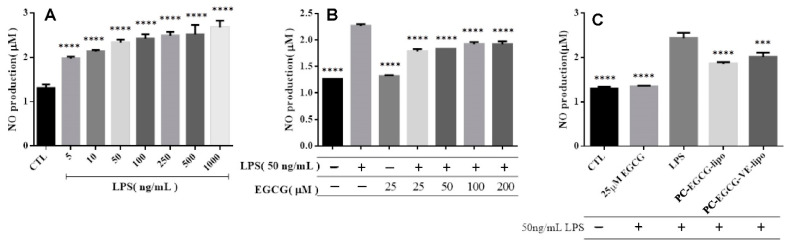
Nitric oxide (NO) production from BV-2 cells (*** *p* < 0.001; **** *p* < 0.0001, *n* = 3). (**A**) Inflammation induced by various concentration of LPS (compared with control group), (**B**) cells treated with EGCG followed by LPS induction (compared with LPS (+) and EGCG (−)), (**C**) cells treated with EGCG, PC-EGCG-liposomes, and EGCG-VE-liposomes then LPS (compared with LPS (+)).

In Figure 3A, the NO release from BV-2 cells induced with 5–1000 ng/mL LPS during 24 h incubation was statistically significant compared to the NO release from the control group. The concentration of LPS used for activation of cell inflammation to work was determined to be 50 ng/mL.

NO release from BV-2 cells treated with 25 μM EGCG was not statistically significant compared to the control group, as shown in Figure 3B. However, the cell inflammation induced with LPS for 24 h showed a significant increase compared to the control group. Those cells treated with 25–200 μM EGCG for 1 h and then activated with LPS showed a statistically significant decrease compared to the group of cells activated with LPS only. NO release did not decrease when EGCG increased from 50–200 μM since the cell viability decreased when EGCG had risen from 50–200 μM, according to Figure 1A.

The NO production of the cells treated with 25 μM EGCG followed by the inflammation induced with 50 ng/mL LPS was not statistically significant compared with the control group (Figure 3C). However, the NO released in the group of cells treated with PC-EGCG-liposomes or PC-EGCG-VE-liposomes followed by LPS activation with 50 ng/mL showed a significant decrease compared to the group of cells treated only with LPS. The NO release from cells pretreated with PC-EGCG-liposomes or PC-EGCG-VE-liposomes was higher than the NO release from the group of cells pretreated by EGCG, which should be explained by the slow release of EGCG from the liposomes.

#### 2.2.4. Cytokine Analysis

**Figure 4:**
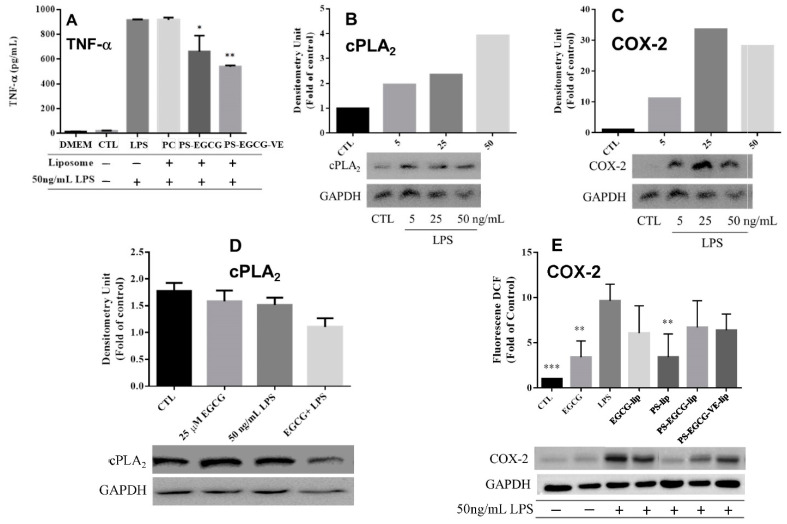
In LPS-induced BV-2 cells, expression of (**A**) TNF-α (compared with the liposome (−) and LPS (+) group, * *p* < 0.05; ** *p* < 0.01, *n* = 3), (**B**) cytosolic phospholipase A2 (cPLA_2_), and (**C**) cyclo-oxygenase-2 (COX-2). In BV-2 cells pretreated by EGCG or liposomes and then induced by LPS, expression of (**D**) cPLA_2_ and (**E**) COX-2 (compared with LPS (+) group ** *p* < 0.01, *** *p* < 0.001, *n* = 3).

In Figure 4A, the concentration of TNF-α of cells treated with LPS after 24 h showed a statistically significant increase compared to that of the control group or the cell culture medium (DMEM). Placebo PC-liposomes were close to that of cells treated with LPS. However, the TNF-α concentration in the group of cells pretreated with PS-EGCG-liposomes or PS-EGCG-VE-liposomes followed by the LPS activation showed a statistically significant decrease compared to that of cells treated with LPS. This decreasing concentration of TNF-α indicates that EGCG-loaded liposomes can reduce the activation of microglial cells induced by LPS. The inhibitory effect of PS-EGCG-VE-liposomes was better than that of PS-EGCG-liposomes.

The phospholipids on the cell membrane can be hydrolyzed by cytosolic phospholipase A2 (cPLA_2_) to produce arachidonic acid. The cyclo-oxygenase (COX) is a key enzyme that converts arachidonic acid to prostaglandin. COX-2 and cPLA_2_ are often generated from inflammation or malignant disease [31,32,33,34]. In Figure 4B, the inflammation was induced by LPS of 5–50 ng/mL for 24 h, and the expression of cPLA_2_ increased when the LPS concentration increased. Figure 4C showed the increased activities of COX-2, induced by LPS (5–50 ng/mL) for 24 h. The expression of COX-2 increased from 5–25 ng/mL LPS, while it decreased at 50 ng/mL. In Figure 4D, the cPLA_2_ expression was reduced when BV-2 cells were pretreated with EGCG and induced by LPS. The expression of COX-2 when LPS activated BV-2 cells was increased compared to the control group (Figure 4E). There was a significant decrease of COX-2 in the group of cells pretreated with EGCG, placebo PS-liposomes, PS-EGCG-liposomes, and PS-EGCG-VE-liposomes, followed by LPS inflammatory induction. Especially, expression of COX-2 of cells pretreated with placebo PS-liposomes had a statistically significant difference from that of cells induced by LPS.

### 2.3. In Vivo Animal Test

#### 2.3.1. Animal Behavioral Test

**Figure 5:**
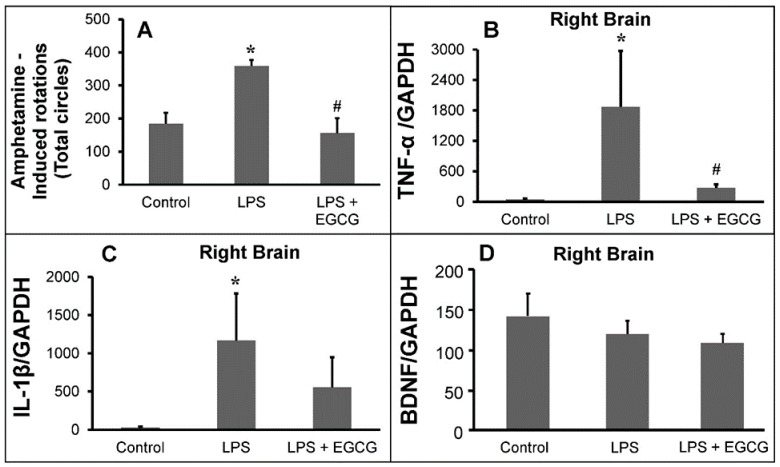
Animal study analysis; (**A**) effects of epigallocatechin-3-gallate (EGCG) liposomes on Parkinsonian syndrome rat rotation behavioral test (*n* = 5 for the control group (injected with 5 µL PBS); *n* = 4 for the LPS-induced group (with 15 μg LPS); *n* = 5 for the LPS-induced and then the PC-EGCG-VE-liposome improvement group (with 15 μg LPS and PC-EGCG-VE-liposomes with 25 μM EGCG), for mean ± SEM, * *p* < 0.05 represents the comparison between the control group and LPS. No difference between the control group and the LPS+EGCG group, # *p* < 0.05 represents the comparison between the LPS group and the LPS+EGCG group). Expression of (**B**) TNF-α, (**C**) IL-1β, and (**D**) brain-derived neurotrophic factor (BDNF) in substantia nigra area of rats sacrificed after rotation behavioral test, *n* = 3, for mean ± SEM, (* *p* < 0.05 represents the comparison between the control group and LPS. No difference between the control group and the LPS+EGCG group, # *p* < 0.05 represents the comparison between the LPS group and the LPS+EGCG group).

The number of circles completed by the Parkinsonian rats after amphetamine administration (shown in Figure 5A) was significantly increased compared to the number of circles completed by the control group rats. Parkinsonian rats′ behavior treated with the various formulation of EGCG was similar to the control group rats. These data indicated that EGCG attenuated LPS-induced unilateral lesions of the nigrostriatal system.

#### 2.3.2. Inflammatory Markers Analysis

The ratio of TNF-α/GAPDH in the treated group was significantly lower compared to the ratio found in syndromic rats. The IL-1β trend was similar to the TNF-α trend.

The in vivo results are consistent with the results of in vitro studies. The expression of brain-derived neurotrophic factor (BDNF) in the treated group was similar to the LPS-induced group (Figure 5D). However, this outcome could indicate that the improvement of limb coordination in Parkinsonian syndrome rats by PC-EGCG-loaded liposomes is caused by reducing neuroinflammation response, but not by increasing expression of BDNF.

It also fits previous reports that reduction of TNF-α and NO production by pretreatment of rats with EGCG (10 mg/kg) for 24 h and induction with LPS after 7 days decreased comparing to that of LPS-treated rats, and concluded that EGCG has a potential therapeutic effect for LPS-induced neurotoxicity due to reduction of TNF-α and NO release.

## 3. Discussion

In this study, the purity of EGCG extracted from green tea was detected to be 90.5%, and the free radical scavenging activity of EGCG was more extensive than 80% within 3 min. It was increasing with a higher concentration of EGCG or longer reaction time. The particle size of PC-EGCG-VE- and PS-EGCG-VE-liposomes were 161.5 and 142.9 nm, which were smaller than that of EGCG loaded in PLGA microspheres [28] with additional β-cyclodextrin (ranging from 1–14 µm). The encapsulation efficiency of PC-EGCG-VE- and PS-EGCG-VE-liposomes were 60.2% and 76.8%, respectively. These results showed that PS-containing liposomes were smaller, more stable, and had higher encapsulation efficiency due to the charge on PS and resulted in a repulsive force between liposomes to avoid aggregation.

Expression of TNF-α in cells pretreated with PS-EGCG- and PS-EGCG-VE-liposomes and then induced by LPS had statistically significant differences compared with that of cells induced with LPS, and similar results were observed in the pretreatment of EGCG on LPS-induced TNF-α expression in BV-2 cells [25] and human macrophages [26]. In summary, the cell morphology and expression of TNF-α of the group treated with EGCG showed an inhibition effect of the inflammation induced by LPS.

In the present study, EGCG pretreatment can reduce the release of NO. Suppression of NO production from LPS-induced BV-2 cells by pretreatment of EGCG-loaded PLGA microspheres was also investigated in our previous study [28].

In the present study, Parkinsonian syndrome in rats is created by the damage to the unilateral substantia nigra region induced by LPS. Another important finding is that, through statistical quantitative analysis, EGCG-loaded liposomes can alleviate the syndrome due to damage to the unilateral midbrain nigrosome region of the rat brain induced by LPS in the rotational test, and production of neuroinflammatory factors TNF-α in the substantia nigra area of the rat brain can also be reduced by EGCG-loaded liposomes. This study indicates that improvement of limb coordination and reduction of neuroinflammation in LPS-induced Parkinsonian syndrome is caused by locally administering EGCG-loaded liposomes but not by increasing expression of BDNF. However, the anti-neuroinflammation results are necessary for a neuroprotective effect [35]. BV-2 activation releases pro-inflammatory factors, which are neurotoxic and lead to cell damage. By preventing BV-2 activation, EGCG provides a neuroprotective effect. Some post-treatment of EGCG for improving proliferation, survival rate, and neuronal differentiation of adult neural stem cells in dentate gyrus induced by LPS indicates that EGCG may be a potential therapeutic agent for neuroinflammatory diseases [27].

Previous pharmacokinetic studies showed that exogenous PS can cross the blood-brain barrier (BBB), in which it appears to have an affinity for the hypothalamus [6], and the oral administration results in peak levels in 1–4 hours. In addition, it was found that PS-containing liposomes mimic apoptotic cells to promote the secretion of anti-inflammatory mediators, such as transforming growth factor-β1 (TGF-β1) (to downregulate NO produced from macrophages) [36] and prostaglandin E_2_ (PGE_2_) by macrophages and microglia cells in vitro [6,37], and also promote the alleviation of inflammation in vivo [38]. Accordingly, PS-containing EGCG-loaded liposomes demonstrated in this study have the advantage of smaller particle size, higher encapsulation efficiency, and inhibiting activation of Parkinsonian syndrome both in microglia cells and in the in vivo rat model, showing an improving anti-inflammatory function and neuroprotection.

The limitation is that our study was carried out with some missing analyses, such as in the case of the neuroprotective effect, and the NeuN staining was not investigated. We also analyzed only BDNF as a neurotrophic factor. FGF2 and IGF2 are also neurotrophic factors that should be considered [39].

## 4. Material and Methods

### 4.1. The Source of Epigallocatechin-3-Gallate

#### 4.1.1. Epigallocatechin-3-Gallate Extraction

The extraction process and purity measurement of extracted EGCG had been published elsewhere [40]. Briefly, we dispersed five grams of green tea powder (Ten Ren Tea Co., Ltd., Taiwan) in 65% ethanol and refluxed at 100 °C for 15 min. The rotary evaporator (N-1000, Eyela, Tokyo, Japan) was used to remove ethanol. We then purified the dried solid in two consecutive extraction processes. The solid dispersed in distilled water was extracted against an equal volume of chloroform. The resulting water phase was then extracted against ethyl acetate. Next, the extracts were obtained from an ethyl acetate phase followed by rotary evaporation and then stored in the containers at low humidity before use.

#### 4.1.2. Free Radical Scavenging Activity Analysis

Most in vitro antioxidant activity evaluation uses 2,2-diphenyl-1-picrylhydrazyl (DPPH) to determine the ability of antioxidants for scavenging free radicals [41]. A total of 200 μM DPPH solution dissolved in methanol (80%, HPLC, Sigma-Aldrich, USA) was prepared, then mixed with EGCG solutions at various concentrations from 0.0625 to 1 mg/mL dissolved in H_2_O (Purelab, ELGA LabWater), respectively. The absorbance was measured at 517 nm using a UV/VIS spectrophotometer (Shimadzu cooperation, Japan). The DPPH scavenging activity of EGCG was calculated using Equations (1).

(1) $$\mathrm{DPPH}\;\mathrm{scavenging}\;\mathrm{activity}\;\left(\%\right)=\left(1-\frac{\mathrm{absorbance}\;\mathrm{of}\;\mathrm{DPPH}\;\mathrm{related}\;\mathrm{with}\;\mathrm{catechin}\;\mathrm{exact}}{\mathrm{absorbance}\;\mathrm{of}\;\mathrm{DPPH}}\right)\times 100\%$$

### 4.2. Liposome Preparation

Solutions for the fabrication of liposome dosage form including 10 mg/mL of L-α-phosphatidylcholine (PC), phosphatidylserine (PS), and cholesterol (CH) solutions dissolved in chloroform, 43.6 mM EGCG solution dissolved in 1 mL H_2_O, and 10 mg/mL α-tocopherol (vitamin E, VE) solution dissolved in ethanol were prepared and then stored in a 4 °C refrigerator.

PC, PS, CH, and VE solutions were mixed in the molar ratio listed in Table 1 to a total volume of 1 mL and 3 μL PKH-26 cell linker kit (Sigma-Aldrich) for cellular membrane labeling fluorescent dye was added and mixed homogeneously. The organic solvent was then removed by a rotary evaporator and a vacuum dryer to obtain a layer of translucent lipid film. In preparation for placebo PC- and PS-liposomes, 1 mL H_2_O was added, and for PC-EGCG-, PS-EGCG-, PC-EGCG-VE-, and PS-EGCG-VE-liposomes, 0.5 mL H_2_O and 0.5 mL EGCG solution was added to the lipid film. The solution was shaken by a vortex mixer at high speed for 10 min to obtain large unilamellar vesicles. A mini-extruder (Avanti Polar Lipid Inc., Alabama, USA) with two polycarbonate filter membranes with a pore size of 400 and 200 nm was used to squeeze the sample 11 times repeatedly. Thus, the liposome was forced to pass through the filter membrane and self-assembled to obtain liposomes with uniform particle size.

#### Analysis of Fabricated Liposome

Particle size and particle size distribution (Polydispersity index, PDI) of liposomes were analyzed by a dynamic light scattering (DLS) apparatus (Zetasizer 3000 HSA, Malvern, U.K.) at 25 °C, and the light scattering angle was 90°. To determine the amount of EGCG encapsulated in liposomes, EGCG-loaded liposomes were placed in a sealed dialysis membrane (MWCO = 1000). The membrane was placed in an isotonic aqueous solution at 4 °C for removing the EGCG molecule that was not encapsulated. Then, dialyzed liposomes and ethanol were reacted at 4 °C for 20 min for rupturing liposomes, and the solution was centrifuged at 10,000 rpm for 20 min at 4 °C in a high-speed centrifuge. The supernatant of the centrifuged solution was collected, and the amount of EGCG loaded in liposomes was estimated by comparing its absorption value at 274 nm to that of the calibration curve. The encapsulation efficiency of EGCG in liposomes was calculated by Equations (2). Surface morphology and liposome structure were observed with a negative staining transmission electron microscope (TEM) using phosphotungstic acid (PTA) dye because liposomes are transparent and colorless.

(2) $$\mathrm{Encapsulat}\;\mathrm{ion}\;\mathrm{efficiency}\;\left(\%\right)=\left(\frac{\mathrm{Total}\;\mathrm{amount}\;\mathrm{of}\;\mathrm{encapsulat}\;\mathrm{ed}\;\mathrm{EGCG}}{\mathrm{Total}\;\mathrm{amont}\;\mathrm{of}\;\mathrm{EGCG}\;\mathrm{in}\;\mathrm{supernatan}\;t}\right)\times 100\%$$

In this study, the concentration of EGCG in EGCG-loaded liposomes was calculated from 43.6 mM (the EGCG stock solution) × 0.5 mL/1 mL × corresponding encapsulation efficiency × the dilution factor in use. All the concentrations describing the EGCG-loaded liposomes in this study denoted the concentration of EGCG in EGCG-loaded liposomes.

### 4.3. In Vitro Cell Analysis

#### 4.3.1. Cell Culture

BV-2 cells were used for in vitro cell experiments. We cultured the cells with a culture medium of Dulbecco′s Modified Eagle Medium (DMEM) (Gibco, Grand Island, NY, USA), supplemented with 2.2 g sodium bicarbonate, 4.8 g HEPES (4-(2-hydroxyethyl)-1-piperazineethanesulfonic acid) buffer adjusted at pH 7.2–7.4, antibiotic-antimycotic 100× (Biowest, France), L-glutamine, and 10% (*v*/*v*) of fetal bovine serum (FBS) (Gibco, Grand Island, USA). The BV-2 cells were cultured in 6-well plates at the density of 10^5^ cells/cm^2^ and incubated under 5% CO_2_ at 37 °C. The activation was induced by lipopolysaccharide (LPS).

The shape of BV-2 cells was round and irregular spindle when they were not stimulated and stimulated by LPS.

#### 4.3.2. Cell Viability

EGCG or EGCG-loaded liposomes with different concentrations and LPS solution were added to the BV-2 cells and incubated for 24 h. The medium was then removed, rinsed once with PBS, and replaced by the medium containing the 3-(4,5-dimethylthiazolyl-2)-2, 5-diphenyltetrazolium bromide (MTT) solutions (3-(4,5-dimethylthiazol-2-yl)-2,5-diphenyl tetrazolium bromide, ab146345, Lot. No. GR3203144-11, Abcam, Cambridge, MA, USA) at the volume ratio of 9: 1. After incubating for three hours, the medium of each well was removed, and dimethyl sulfoxide (DMSO) was added to dissolve the purple crystals for 5 min. The enzyme-linked immunosorbent assay (ELISA) reader (Multiskan FC, Thermo Scientific, Shanghai, China) was used to detect the absorbance at 570 nm of each well. The cell viability was calculated using Equations (3).

(3) $$\mathrm{Cell}\;\mathrm{viability}\;\left(\%\right)=\left(\frac{\mathrm{Absorbance}\;\mathrm{of}\;\mathrm{cells}\;\mathrm{cultured}\;\mathrm{with}\;\mathrm{EGCG}}{\mathrm{Absorbance}\;\mathrm{of}\;\mathrm{control}\;\mathrm{group}}\right)\times 100\%$$

#### 4.3.3. Cell Morphology

BV-2 cells were cultured in a 6-well plate (10^5^ cells/ cm^2^) for 24 h to stabilize cell attachments. We established various culture conditions to monitor cell morphology: (i) cells treated with 25 µM EGCG; (ii) cells treated with 50 ng/mL LPS; and (iii) cells pretreated with EGCG for 1 h, followed by 50 ng/mL LPS treatment. The inverted microscope (Nikon Eclipse 50i, Tokyo, Japan) was used.

#### 4.3.4. Nitric Oxide Release

We performed the nitric oxide (NO) release assessment by seeding the cells with various concentrations of EGCG-loaded liposomes in one hand, and on the other hand, cells were seeded without EGCG. After 24 h incubation, cells were treated with 50 ng/mL LPS for both conditions and incubated for 24 h. Next, we collected 50 µL of culture media from each culture condition and transferred them into 96-well plates. 50 µL of Griess reagent was used to evaluate NO concentration in cells and was then added into different wells. The disposal was protected from light and incubated at 25 °C for 20 min. ELISA reader assessed the optic density (OD) values at 540 nm wavelength for determining the concentration of NO released by cells.

#### 4.3.5. Cytokine Analysis

##### TNF-α Assessment

We cultured BV-2 cells in 6-well plates. Cells were then treated with 125 μg/mL EGCG-loaded liposomes or placebo and incubated for one hour. We induced the inflammation by treating the cells with 50 ng/mL LPS and incubating them for 24 h. The culture media were collected for TNF-α secreted assessment using TNF-α mouse ELISA assay kit (R&D Systems’ biotechnology brand).

##### COX-2 and cPLA2 Analysis by Western Blotting

To evaluate the COX-2 and cPLA2 secretion, BV-2 cells were in 6-well plates as aforementioned. When the cells reached 90% confluently, the medium was discarded. We then collected the cells in cold conditions to avoid protein degradation. We lysed the cell by adding 100 µL lysis buffer and protease inhibitor into them at 4 °C for 45 min. The tubes were centrifuged at 15,000 rpm for 15 min at 4 °C, and the supernatant was collected and stored for further analysis. The protein content was measured with a Pierce bicinchoninic acid protein assay kit (Pierce Biotechnology). Proteins (20 μg) from each sample were suspended in Laemmli buffer, heated for 5 min at 100 °C, and separated by sodium dodecyl sulfate-polyacrylamide gel electrophoresis (SDS-PAGE) on a 4~12% gradient. Proteins were electroblotted onto a hydrophobic polyvinylidene difluoride membrane (Pall Corporation, Port Washington, NY, USA). Following transfer, membranes were blocked in 5% bovine serum albumin (BSA) in Tris-buffered saline solution (TBST) with 0.1% Tween-20 for 45 min and then incubated overnight at 4 °C with a primary anti-cyclo-oxygenase (COX)-2 antibody (GTX100656, diluted 1:1000; GeneTex, Alton Pkwy, CA, USA) and a primary anti-cPLA2. After washing three times with 1x TBST, the membranes were probed with a secondary antibody (horseradish peroxidase (HRP)-conjugated anti-rabbit immunoglobulin G (IgG)) (GeneTex) for one hour and washed three times with TBST. Immunoreactivity was detected using an enhanced chemiluminescence (ECL) kit (GE Healthcare, Chicago, NY, USA) and visualized with the UVP system (Analytic Jena, Upland, CA, USA). Anti-GAPDH from Santa Cruz (Heidelberg, Germany) was used as an internal control. Image J software (1.52 k, Wayne Rasband, NIH) was used to quantify the intensity of the protein bands.

### 4.4. Animal Study

#### 4.4.1. Ethics Statement

The animal experiment in the current study was conducted following the procedures approved by the Institutional Animal Care and Use Committee (IACUC) of Chung Yuan Christian University under project number 106011 (accessed on 10 August 2017).

#### 4.4.2. Rat management

**Figure 6:**
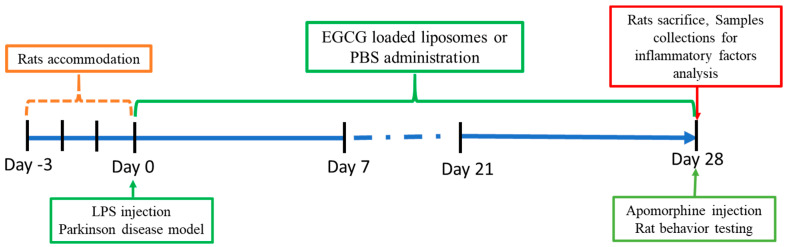
Experimental timeline of Parkinson’s disease model in rats treated with EGCG-loaded liposomes (or PBS).

A total of 14 male Sprague Dawley (SD) rats were purchased from BioLASCO Taiwan Co., Ltd. The weight of the rats was around 250 to 300 g. The animals were divided into three groups, as indicated in Table 2. We performed the experiments according to the timeline in Figure 6.

The LPS-induced PD rat model, which injected LPS unilaterally into the substantia nigra, which can elicit strong microglia-mediated neuroinflammation that is followed by the specific death of nigral dopaminergic neurons, is widely used to study the inflammatory process in the pathogenesis of PD [42,43], and the anti-inflammatory mechanism of some substances. In our experiment, LPS was injected into the substantia nigra of the right midbrain. The LPS injection led to neuroinflammation by destroying the dopamine neuropathway of the substantia nigra. The EGCG-loaded liposome was administered for four weeks to investigate its neuroprotective activity. The rotation behavior of rats and the expression of neuroinflammatory markers (TNF-α, IL-1β) and brain-derived neurotrophic factor (BDNF) have been evaluated.

#### 4.4.3. Parkinsonian Syndrome Rat Model

The rats were anesthetized intraperitoneally with 0.06 mL/10 g of Zoletil 50 (50 mg/mL) dilute 5X (1 mL Zoletil in 0.1 mL Rompun 2% and 3.9 mL normal saline). The rats were then fixed in a stereotactic position. The rats’ hair was shaved off, they were disinfected with povidone-iodine, and the skull was exposed. We injected into the right substantia nigra 3 μL LPS (5 μg/μL) (from *Escherichia coli* 0111:B4, Sigma, St. Louis, MO, USA). To achieve the nigrostriatal system′s unilateral lesion, a microsyringe was used with a flow rate of 1 μL/min. The needle was held in the head for 7 min to prevent reflux of the drug. The skin was sutured for observation.

#### 4.4.4. Animal Behavioral Test

Administration of apomorphine induced abnormal contralateral rotations in the PD model rats [44]. Amphetamine (2 mg/kg) was administered to the rats after four weeks. The rotation toward the side of injury was observed. For the control group, we injected 5 μL PBS in the unilateral brain substantia nigra area. The rat was placed into a 40 cm-diameter bowl, and the counterclockwise (contralateral) rotations were monitored. The image was recorded within 1.5 h to count the number of rotations, in which a circle is defined as a rotation of 270 ° of the rat’s head. The damage was proportional to the number of rotations.

#### 4.4.5. Animal Inflammatory Factor Analysis

Rats after the rotational test were anesthetized, sacrificed, and perfused. The brain’s damaged nigrostriatal region was collected for RNA extraction with 1 mL TRIzol^®^ (Invitrogen, Carlsbad, CA, USA) reagent according to the manufacturer’s instruction. The RNA concentration was measured using a micro-volume spectrometer. Real-time reverse transcription-polymerase chain reaction (qRT-PCR) was performed by SuperScript III First-Strand Synthesis SuperMix and analyzed by 7300 Real-Time PCR System (Applied Biosystems, USA). cDNA was generated by reverse transcription of 1 μg RNA with reverse transcriptase (RT) and oligo(dT) primers (Table 3). The cDNA was diluted to 2.5 ng/μL, and cDNA, primers (Table 3), and FastStart Universal SYBR^®^ Green Master (Rox) (Roche, Germany) were added to a qPCR 8-strip tube for the qPCR reaction.

### 4.5. Statistical Analysis

All experimental data were expressed as mean ± standard deviation (mean ± SD). Data analysis was performed using Excel and GraphPad Prism software version 6.0 (GraphPad Software, La Jolla, CA, USA). The statistical analyses were T-test and one-way or two-way ANOVA with the component of Dunnett’s multiple comparisons test. A *p*-value less than 0.05 was considered a statistically significant difference (SSD) between the experimental and control groups.

## 5. Conclusions

To improve the low oral bioavailability of catechins, EGCG was loaded in liposomes in this study. It is found that PS-containing liposomes were smaller and more stable. It has higher encapsulation efficiency, and the addition of vitamin E can protect EGCG from oxidation and improve encapsulation efficiency. In the in vitro study, expressions of TNF-α and NO production from BV-2 cells were all reduced after pretreatment of EGCG-loaded liposomes on LPS-induced BV-2 cells. Therefore, the EGCG-loaded liposomes have played an essential role in the neuroinflammatory response as an inhibitor. The beneficial effects have been to prevent cells from apoptosis in the neuroinflammatory reactions.

In in vivo study, the Parkinsonian syndrome in rats induced by LPS to the unilateral midbrain substantia nigra region has been improved. The neuro-inflammation mechanism, including the TNF-α secretion, has been inhibited by post-treatment of EGCG-loaded liposomes. We demonstrated that EGCG would be a more valuable candidate for treating neurodegenerative diseases by alleviating the inflammation in the brain. The subsequent investigation should focus on the inflammatory pathways by using a lysosome tracker to track the liposome entrance into the cell and the release of EGCG from the liposome.

## Figures and Tables

**Table 1:** Characteristics of the formulation of EGCG-loaded liposomes.

Samples	Molar Ratio				Diameter (nm)	Encapsulation Efficiency(%)	PDI
	PC	PS	CH	VE			
PC-liposome	0.73	-	0.27	-	184.4 ± 1.59	-	0.218
PS-liposome	0.24	0.49	0.27	-	117.47 ± 1.12	-	0.092
PC-EGCG-liposome	0.73	-	0.27	-	155.2 ± 1.23	55.4%	0.121
PS-EGCG-liposome	0.24	0.49	0.27	-	132.86 ± 2.05	70.4%	0.094
PC-EGCG-VE-liposome	0.73	-	0.27	0.07	161.5 ± 0.56	60.2%	0.058
PS-EGCG-VE-liposome	0.24	0.49	0.27	0.07	142.9 ± 0.36	76.8%	0.101

PC: L-α-phosphatidylcholine; PS: phosphatidylserine; CH: cholesterol; VE: vitamin E (α-tocopherol); EGCG: (−)-epigallocatechin-3-gallate; PDI: polydispersity index.

**Table 2:** Rat groups and treatment administered.

Experimental Groups of Rats	LPS Injected 3 μL (5 μg/μL)	EGCG-Loaded Liposomes 2 μL (12.5 μM)	PBS 5 μL	Amount
Rats with Parkinson Disease syndrome	Yes	No	Yes	4
Rats with Parkinson Disease syndrome treated	Yes	Yes	No	5
Control group	No	No	Yes	5

**Table 3:** Primer sequences used for qRT-PCR analysis.

Gene	Forward Primers (5′→3′)	Reverse Primers (5′→3′)
*TNF-α*	TGA CTC GTG GGA TGA TGA CG	CTG GAG ACT GCC CAT TCT CG
*IL-1β*	CTC ACA CTC AGA TCA TCT TCT C	GGT ATG AAA TGG CAA ATC GG
*BDNF*	GGT CAC AGC GGC AGA TAA AAA G	TTC GGC ATT GCG AGT TCC AG
*GAPDH*	GA AGA GAG AGG CCC TCA G	TGT GAG GGA GAT GCT CAG TG

## Data Availability

The data presented in this study are available in this article *Int. J. Mol. Sci.* **2021**, *22*, x or Supplementary Materials here.

## Supplementary Materials

The following are available online at https://www.mdpi.com/1422-0067/22/6/3037/s1.
