# Supplementary material for: Epigallocatechin-3-Gallate-Loaded Liposomes Favor Anti-Inflammation of Microglia Cells and Promote Neuroprotection
Source: Int J Mol Sci. 2021 Mar 16;22(6):3037. doi: 10.3390/ijms22063037 (PMC8002297; doi:10.3390/ijms22063037)
Supplement: Supplementary file 1 [file ijms-22-03037-s001.pdf]

International Journal of
Molecular Sciences

MDPI

Green Tea Extract Loaded Liposomes for Anti-inflammation of Microglia Cells Induced by Lipopolysaccharide

Chun-Yuan Cheng [1,2], Lassina Barro [2], Tai-Wei Feng [2,4], Xiao-Yu Wu [2] Che-Wei Chao [3], Ruei-Siang Yu [2], Ting-Yu Chin [3,*], Shang-Ting Tsai [2,4,†,*],Ming Fa Hsieh [2,4,*]

Supplementary Data

1. EGCG extract analysis

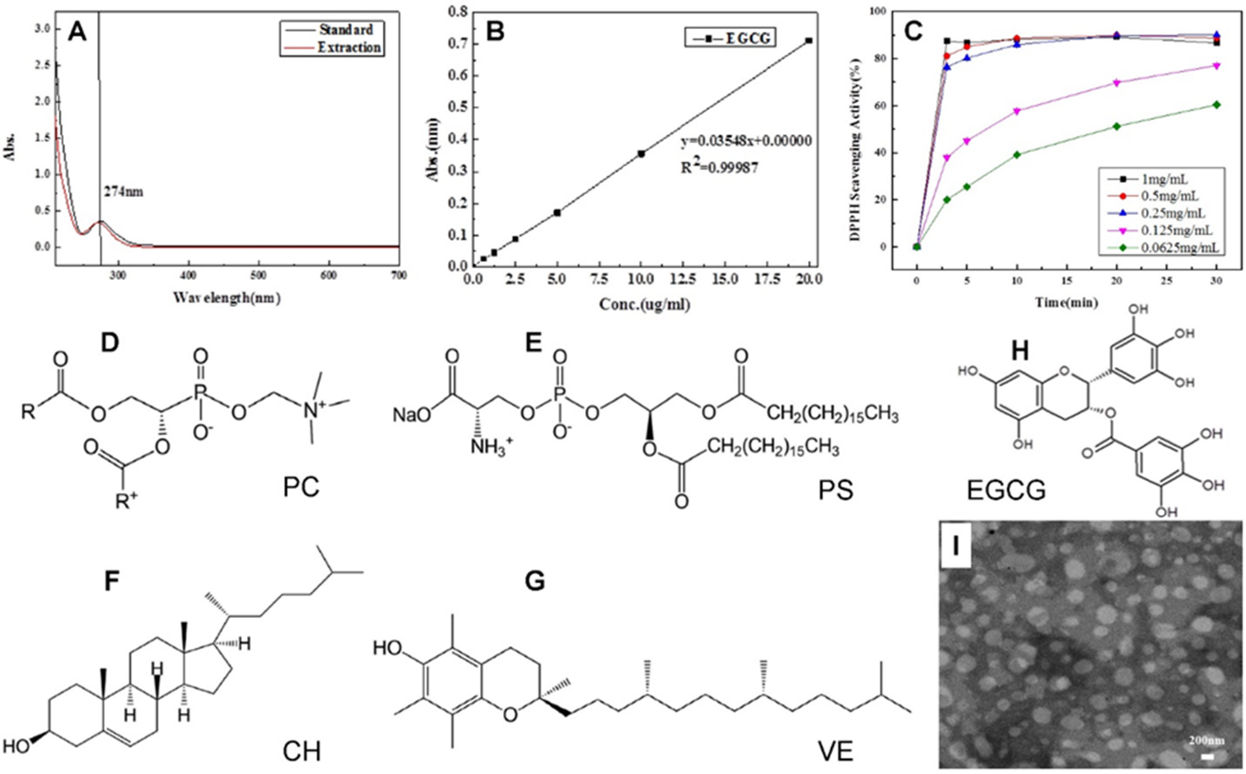

Figure S1. (A) UV-VIS spectrum of EGCG extract and standard. (B) Calibration curve of EGCG. (C) DPPH scavenging activity post adding EGCG. (D)-(H) Structures of (D) L-α-phosphatidylcholine (PC), (E) phosphatidylserine (PS), (F) cholesterol (CH), (G) vitamin E (α-tocopherol, VE), and (H) (−)-epigallocatechin-3-gallate (EGCG). (I) TEM of EGCG liposome, the scale bar was 200 nm.

The spectra of extract and standard were nearly coincident, and the maximum absorbance of both was 274 nm [31]. The purity of EGCG extract was 90.5% calculated from the absorbance at 274 nm of 0.5 mg/mL according to the calibration curve shown in Figure S1B.

1.1 Free radical scavenging activity analysis

In Figure S1C, free radical scavenging activities of EGCG extract of 1, 0.5, and 0.25 mg/mL were more extensive than 80% at 5 min, and all concentrations were more extensive than 50% at 30 min.

1.2 Morphology analysis

Figure S1I shows the TEM image of EGCG liposome at 100 kV and magnification of 50 k, in which the lighter and darker areas showed hydrophobic and hydrophilic groups

of liposomes. The liposome shape was round, and particle size was less than 200 nm, indicating that liposomes can be self-assembled in an aqueous solution.

2. Uptake of liposomes in activated cells

In Figure S2 A-C, the morphology of BV-2 was spindle-shaped when activated with 50 ng/mL LPS. In Figure S2 D-F, when BV-2 cells were pretreated with placebo PC-liposome for one hour and then induced by LPS, PC-liposome was not taken up by cells. In Figure S2 G-O, when BV-2 cells were treated with PKH-26 dye stained placebo Ps-, PS-EGCG- and PS-EGCG-VE-liposomes for one hour and induced by LPS, PS-EGCG- and PS-EGCG-VE-liposomes were tracked by fluorescence microscopy and taken up by BV-2 cells (Figure S2 L and O).

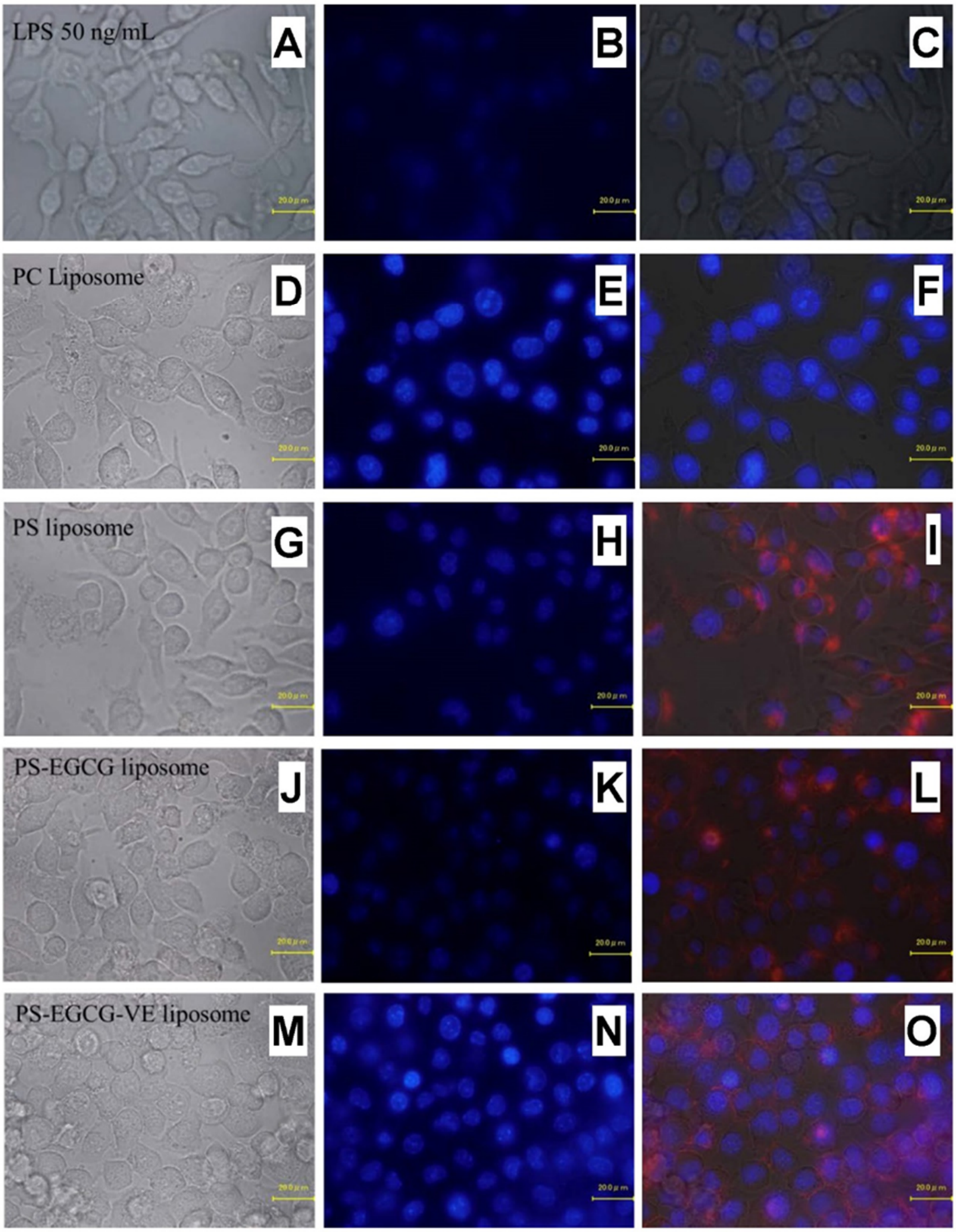

Figure 2. Uptake of liposomes in LPS activated microglial BV-2 cells induced with (**A**)–(**C**) LPS, pretreated with (**D**)–(**F**) PC-, (**G**)–(**I**) PS-, (**J**)–(**L**) PS-EGCG-, (**M**)–(**O**) PS-EGCG-VE-liposomes and then induced with LPS. Cells of (**B**), (**E**), (**H**), (**K**) and (**N**) were stained by Hoechst stains as nuclear localization. Cells of (**I**), (**L**) and (**O**) were treated with PKH-26 dye stained PS-, PS-EGCG- and PS-EGCG-VE-liposome respectively.

References

[1] J.S. Al-Amri, M.M. Hagras, I.M. Mohamed, Effect of epigallocatechin-3-gallate on inflammatory mediators release in LPS-induced Parkinson's disease in rats, *Indian J. Exp. Biol.* **2013**, *51*, 357–362.

[2] L. Hritcu, L.D. Gorgan, Intranigral lipopolysaccharide induced anxiety and depression by altered BDNF mRNA expression in rat hippocampus, *Prog Neuropsychopharmacol Biol Psychiatry*. **2014**, *51*, 126–132.
